# MMB-GUI: a fast morphing method demonstrates a possible ribosomal tRNA translocation trajectory

**DOI:** 10.1093/nar/gkv1457

**Published:** 2015-12-15

**Authors:** Alex Tek, Andrei A. Korostelev, Samuel Coulbourn Flores

**Affiliations:** 1 Cell and Molecular Biology Department, Uppsala University, Box 596, Uppsala 751 24, Sweden; 2 RNA Therapeutics Institute, Department of Biochemistry and Molecular Pharmacology, University of Massachusetts Medical School, 368 Plantation St., Worcester, MA 01605, USA

## Abstract

Easy-to-use macromolecular viewers, such as UCSF Chimera, are a standard tool in structural biology. They allow rendering and performing geometric operations on large complexes, such as viruses and ribosomes. Dynamical simulation codes enable modeling of conformational changes, but may require considerable time and many CPUs. There is an unmet demand from structural and molecular biologists for software in the middle ground, which would allow visualization combined with quick and interactive modeling of conformational changes, even of large complexes. This motivates MMB-GUI. MMB uses an internal-coordinate, multiscale approach, yielding as much as a 2000-fold speedup over conventional simulation methods. We use Chimera as an interactive graphical interface to control MMB. We show how this can be used for morphing of macromolecules that can be heterogeneous in biopolymer type, sequence, and chain count, accurately recapitulating structural intermediates. We use MMB-GUI to create a possible trajectory of EF-G mediated gate-passing translocation in the ribosome, with all-atom structures. This shows that the GUI makes modeling of large macromolecules accessible to a wide audience. The morph highlights similarities in tRNA conformational changes as tRNA translocates from A to P and from P to E sites and suggests that tRNA flexibility is critical for translocation completion.

## INTRODUCTION

Macromolecular viewers such as Chimera, (1) VMD, (2) PyMOL, (3) and YASARA, (4) are a key tool of structural biology and related fields. Macromolecules are visually complex objects often containing millions of atoms. Key structural details must be combined with context in a way that elucidates functions and mechanisms, thus leading viewers have many user-configurable rendering modes (5). The processing and memory overhead involved with storing and rendering large molecules presented a significant challenge in the early days, and this was overcome with multiscale methods (5). All popular viewers allow simple zoom, translation, and rotation operations. Most also have various means of translating and rotating individual subunits. Pymol even allows moving individual atoms, but with no force field (3).

All-atoms, physics-based Molecular Dynamics (MD) (6) codes such as GROMACS and NAMD are also widely used tools. MD has evolved over several decades, in tandem with massively parallel computer hardware, to predict the motion of very large systems including the ribosome. However, such motions can be modeled on a time scale of single milliseconds with great effort, (7,8) whereas ribosome conformational rearrangements, such as translocation, may occur on the scale of tens to hundreds of milliseconds (7,9–11). Thus, no MD simulation has observed the key translocation completion step (12). In the meantime, the realization that structure is hierarchical (13–16) has led to the rise of multiscale methods (13,17–18) which focus computer power on regions of interest such as hinges and active sites. Many of these methods are fast enough for interactive use, but until now have been available only as command-line packages.

Plug-in extensions have been developed to expand the capabilities of viewers. VMD connects with NAMD to do Interactive MD, though interaction compatible with the human attention span is possible only for small systems. Chimera supports rigid-body fitting (19) of atomic structures to low-resolution density maps. VMD supports fitting of fully-flexible atomic structures to density maps, (20) though for larger molecules this takes too long to be considered interactive (20,21). Chimera's Modeller plugin provides some modeling tasks such as mutating residues and optimizing side chain conformation (22), and homology modeling for proteins (23) (though not RNA) (24,25). There is no prior published viewer that models template-free folding of macromolecules (26,27).

In this work, we create a single interactive graphical environment in which the non-specialist can use MMB to quickly and intuitively perform multiple tasks previously in the domain of computational structural biologists. MMB is a multiscale (28), internal-coordinate (29) macromolecular modeling code (30). It enables the user to fully control the flexibility of the system, at the level of domains, residues or individual bonds (17). One can apply a wide array of constraints and forces, including base-pairing forces (e.g. for RNA folding) (31), springs (e.g. for homology modeling) (24,32), density-based forces (for fitting to density maps) (21), and a conventional MD force field (33,34). Its multiscale aspect is key to its fast processing of large complexes including the ribosome (35,36). Limited *flexibility zones* can be created which include hinge points, interfaces, mutation and active sites, or other regions of interest, while the MD force field can similarly be limited to *physics zones* which surround these regions (34,36,37). Regions which are made rigid become kinematically a single body (17), leading to up to 2000-fold savings in computer time (36). Limiting flexibility in this way can in the case of analyzing point mutations actually *improve* accuracy, since the structural refinement can be limited to regions that need it the most (37).

Morphing is one such task (38,39). A morph is an interpolated trajectory connecting two or more conformations of a macromolecule, with the objective of explaining, relating, or analyzing them. Morphing methods typically only generate a single, directed trajectory (40) rather than the many trajectories that would be needed to compute thermodynamic quantities; thus morphing may complement but not substitute MD. A goal of early morphing methods was simply avoiding gross chemical distortions (steric clashes, covalent bonds of physically improbable geometry) (40). More recently, it was proposed that a physically reasonable morph trajectory should pass near experimentally observed intermediates as evaluated *a posteriori* (an objective measure that we adopt), (41) though there was no suggestion that it needs to be able to discriminate stable from unstable intermediates along the trajectory *a priori*. Directed simulations which *can* compute thermodynamic quantities are referred to as biased MD. Such methods include Steered MD, Umbrella Sampling, Potential of Mean Force, etc. (42).

Some of the goals of morphing can be crudely obtained with a mere superposition of the two experimental endpoint structures, also referred to as toggling. Simple morphs based on linear interpolation can be generated economically in standard viewers, but these create unphysical structures (43) (e.g. containing steric clashes). Normal-mode approaches in cartesian coordinates such as Elastic Network Models are also economical, (44) but produce bond distortion and cannot handle net displacement of subunits. More sophisticated methods often require considerable expertise and computer time. An internal-coordinate approach underlies some of the best methods; (45) iMODS, for example, uses this to maintain bond geometry in normal mode morphing (46). However, many methods cannot handle proteins, RNA, and DNA simultaneously. Some require that the sequences and atom counts of the biopolymers be homogenized (40). To our knowledge, no published method other than MMB (35,36) can handle ribonucleoprotein complexes as large as the ribosome, at all-atom resolution, with insertions (even of entire biopolymers), deletions, substitutions, and translocations, all while avoiding steric clashes. In this article, we compare MMB against five other morphing methods using a benchmark dataset (45). Using this method on a laptop, we generate an all-atom trajectory of tRNA translocation in the 70S ribosome, catalyzed by elongation factor G (EF-G) (∼150 000 atoms). This allows us to visualize interesting transitions between observed states, which are not accessible by existing experimental methods.

## MATERIALS AND METHODS

### A GUI built on the Chimera platform allows users to intuitively control all MMB features

**Figure 1.**
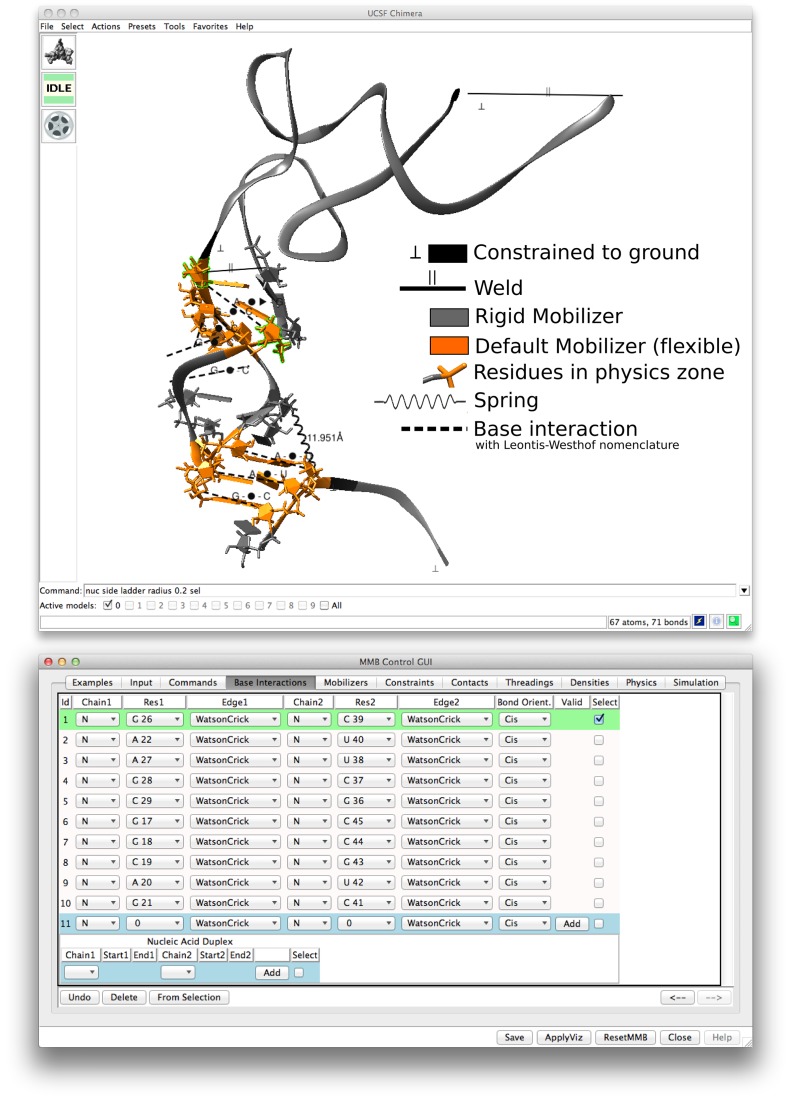
MMB Control GUI and visualization of MMB commands on the 3D model in Chimera. The GUI allows one to specify forces, constraints, flexibility, and run parameters for an MMB job. Related MMB modeling features are organized in tabs. The user can add commands or specify parameters using drop-down lists or text fields, or by selecting objects in the 3D view and clicking the *From Selection* button. Conversely, selecting an object in the GUI highlights the corresponding object in the 3D view. The *ApplyViz* button displays a representation of all the commands on the 3D model with colors and shapes as described in the legend (added on the figure). We here show a model of an mRNA-tRNA interaction.

The main objective of the GUI is to offer an interactive and user-friendly way to input MMB commands and check the output of the simulation. Details on implementation and usage are given in the Supplementary Information and figures. Three steps must be followed to run a simulation. The first step is to initialize MMB with biopolymers (RNA, DNA, and protein chains), represented by a type, chain ID, sequence and structure. Users can specify this in a form, in a command line, or in an input command file; alternatively they can load from a PDB file or Chimera model. The second step is to set the flexibility, constraints, forces, and physics zones, using drop-down lists and other widgets (37). Chimera then displays an intuitive visual representation of the commands; likewise command parameters (e.g. atoms involved in an interaction, residues of altered flexibility, etc.) can be specified using Chimera selection features (Figure 1). The third step is to run a simulation. A form is presented with commonly used parameters, like reporting intervals, temperature *etc*. The user can easily start, stop, and restart the simulation, and review the trajectory.

### Morphing

The conformational states of macromolecules are high-dimensional and subject to thermal motions, and are therefore best defined as an ensemble. Similarly, the trajectory connecting such states is also an ensemble (38). Morphing typically does not attempt to predict the entire ensemble, but only to generate a clash-free, physically rational, trajectory connecting an initial (e.g. open) to a final (e.g. closed) conformation (38). Morphs are useful for classifying motions, (39) and otherwise visually relating conformational states (38). In our implementation, they can be used to transfer conformational information from one ribonucleic complex structure onto another, where the two complexes may have significant sequence differences and be associated with a different number and type of subunits. The root mean square deviation (RMSD) of the trajectory from an experimentally observed intermediate structure, at closest approach, is an objective measure of the quality of the morph (41).

By judiciously adjusting degrees of freedom and regions subjected to a force field, (37) MMB is able to perform fast morphing while maintaining realistic interactions between moving parts at an atomistic level. The first step is to rigidly align the initial and final structures. MMB computes a gapped alignment, using SeqAn, (47) between user-specified biopolymers in the initial and final structures and connects corresponding atoms with springs. These are the same springs used for homology modeling in (34) and rigid alignment in (48).

The user also defines flexible segments in the initial structure, while keeping the final structure rigid. The initial and final structures can comprise protein, DNA, RNA, water, ions, and small molecules, and can have different chain counts. The user can also place collision-detecting spheres at hinges and interfaces, or specify *physics zones* (37) in which an MD force field is active. As in other applications of MMB, defining the flexible regions is crucial. Hinges are often annotated in the literature (21,49) or can be predicted using web servers such as HingeMaster (50). They can also be determined visually and interatively using the morph itself (49). The GUI eases model preparation as users can use Chimera's tools (selection, sequence viewer) to specify the chains to align, flexibility and physics zones, and perform the initial rigid alignment. Driven by the springs, the semiflexible initial structure is then aligned with the final structure. The process can be stopped after a specified simulation time, upon reaching a specified convergence criterion, or by user command.

### Recapitulating intermediate structures with morphing

We tested our ability to recapitulate intermediate structures from the Weiss & Levitt benchmark of morphing methods (45). The five initial structures were submitted to HingeMaster to predict hinge residues. The results were manually filtered to define up to three flexible groups of residues in the structures. We set up the morph simulations with MMB using these data, applying a physics zone (37) of 10Å around all flexible residues. Prior to the simulation, each pair of starting and target structures was rigidly aligned using the Chimera *match* command.

From each trajectory, we computed the *improvement* score described in Weiss and Levitt (45). It measures how much better an interpolated structure is as an approximation of the known intermediate, relative to the initial and final structures:

\begin{equation*} \begin{array}{*{20}l} {improvement = } \\ {\frac{{\min [rmsd(AB),rmsd(CB)] - \min [rmsd(iB)]}}{{\min [rmsd(AB),rmsd(CB)]}} \times 100\% } \\ \end{array} \end{equation*}

where *A*, *B* and *C* are the initial, intermediate and final crystal structures respectively. *i* are the interpolated structures along the morph trajectory. All RMSD values are based on Cα atoms and computed with Chimera. To supplement RMSD, we also report sRMSD in Supplementary Figure S3. sRMSD is computed between two structures by aligning based on one of two domains, then computing RMSD on the other (51). This measure emphasizes the large scale conformational rearrangement.

**Figure 2.**
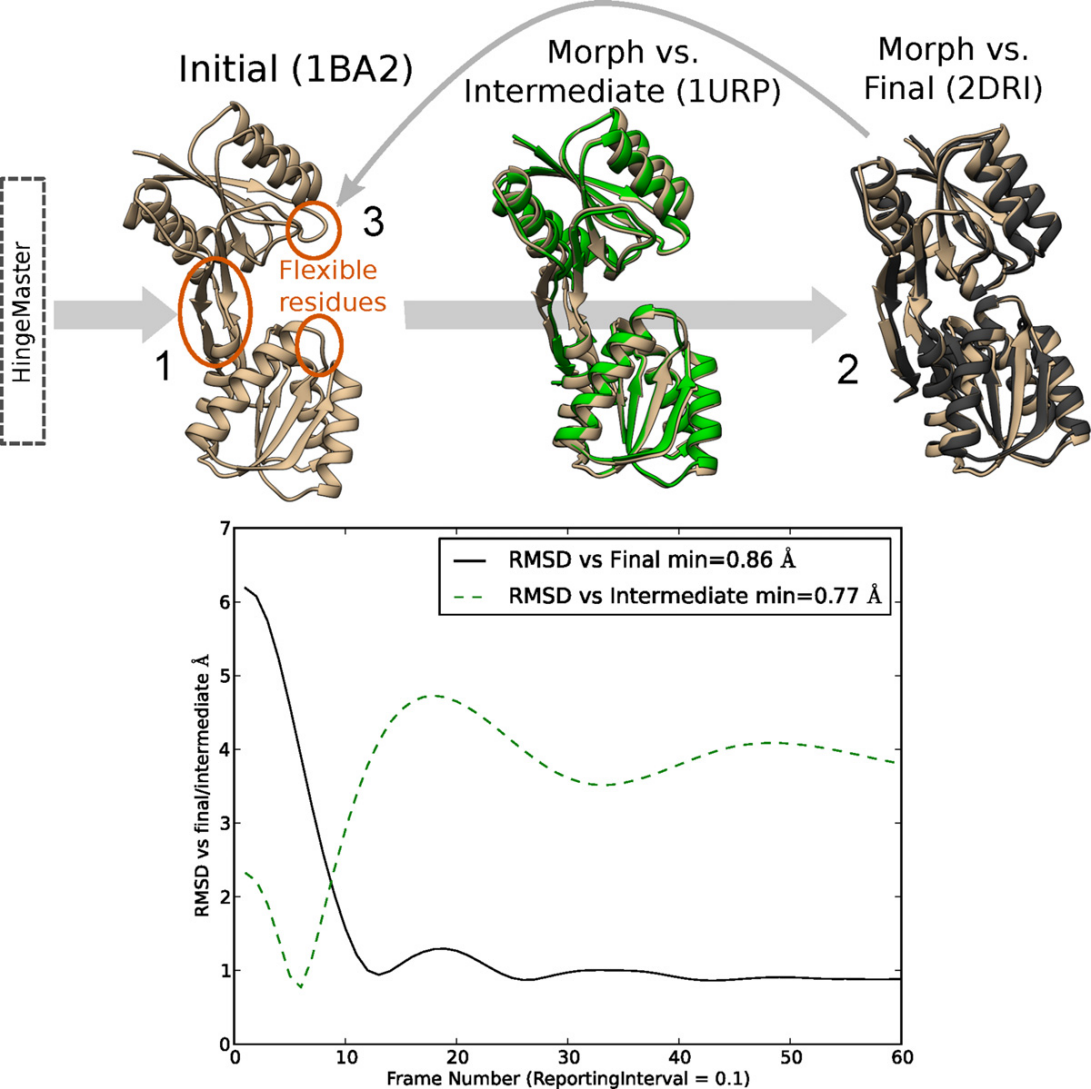
Procedure for morphing and recapitulating an intermediate, with Ribose Binding Protein as an example. 1: Use HingeMaster (or another hinge prediction program, or inspection) to predict interdomain hinges in the initial structure (here PDB ID: 1BA2). Flexibilize these residues. MD forces apply in a small neighborhood about the flexible residues as described in (33,36). 2: Apply springs to connect residues in the thus-flexibilized model to corresponding residues in the final structure (here 2DRI). Allow the model to relax towards the final structure. 3: If the model did not match the final structure, increase flexibility. Here cleft loop (circled) flexibility was needed for closure. Repeat the morph process with the new flexibility scheme. Structure in green is an intermediate (1URP), superimposed with the closest structure along the morph trajectory (tan). Plot: Cα RMSD vs. the intermediate and final structures. Note the very low minimum RMSD (0.77 Å) versus the intermediate.

**Figure 3.**
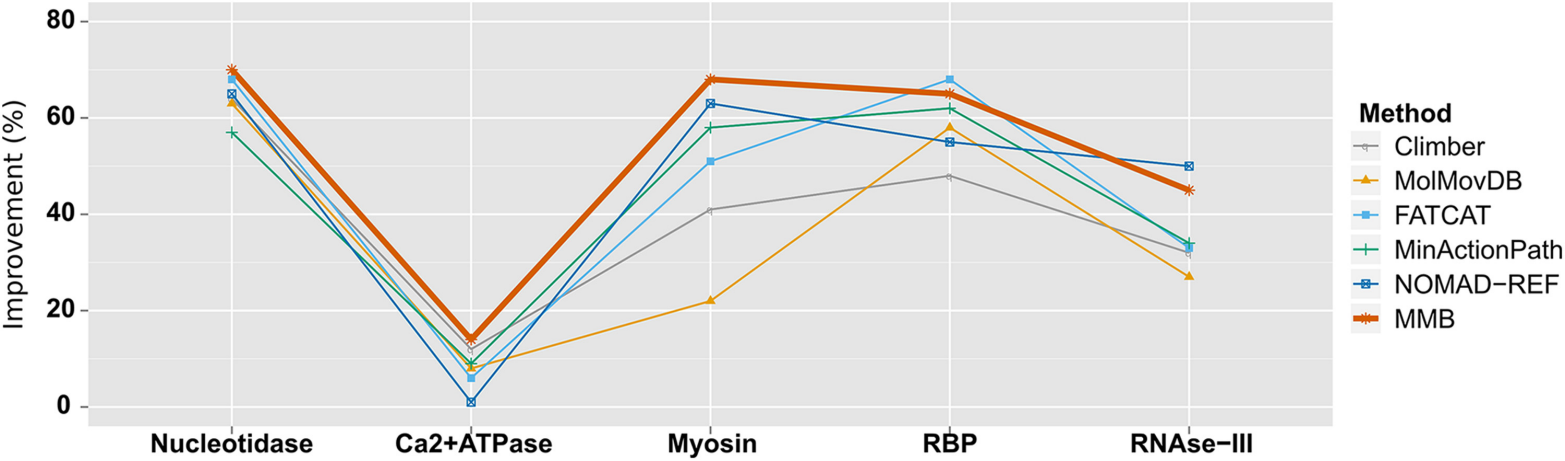
Intermediate structure recapitulation performance of different morphing methods on five protein systems, as described in Weiss and Levitt (45). Improvement scores for MMB were calculated as described in the text. Other values come from Weiss and Levitt (45) and personal communication with D. Weiss.

The results of morphing Ribose-binding protein (RBP) are shown in Figure 2; the complete set of five cases is presented in Supplementary Figure S3. Table 1 and Figure 3 show the improvement scores obtained for each system. It appears that our method gives the highest improvement score in three cases, and is close to the respective best method in the other cases. Concerning performances on Ca2+-ATPase and Rnase III, where MMB ranks first and second, Weiss and Levitt report better scores when using extreme parameters for their method, Climber. They obtained improvement scores of 16% and 48% respectively but with very long simulations. NOMAD-Ref ranked first on RNase III with 50% improvement but produced an erratic trajectory. In contrast, MMB's improvement does not vary dramatically depending on the molecules morphed, demonstrating its robustness. It also produces smooth trajectories, and reaches convergence in a few minutes on a single laptop CPU core (Macbook Air with an Intel Core i5 CPU at 2.8 GHz on OS X 10.8.5).

**Table 1.** Recapitulation of intermediate structures with MMB's morphing feature

Protein	RMSD A versus B	RMSD B versus C	Minimum RMSD morph versus B (Å)	Improvement	Ranking of MMB result versus Weiss and Levitt benchmark	Best improvement from Weiss & Levitt	
5′-Nucleotidase	5.42	4.72	1.42	70%	1	68%	FATCAT
Ca^2+^-ATPase	13.75	10.10	8.67	14%	1^a^	12%	Climber
Myosin	16.56	12.01	3.84	68%	1	58%	NOMAD-Ref
RBP	2.22	4.20	0.77	65%	2	68%	FATCAT
RNase III	7.26	13.15	3.96	45%	2^a^	50%	NOMAD-Ref

The improvement score (%) is defined in the text. In the second and third columns, A = initial structure (e.g. 1QVI for Myosin), B = intermediate (e.g. 1KK7 for Myosin), C = final (e.g. 1KK8 for Myosin), following Weiss and Levitt. Thus, e.g. for Myosin, 3.84 Å is 68% improved compared to 12.01 Å. Rightmost two columns refer to Weiss and Levitt results.

^a^Specific cases discussed in the text.

### Trajectory of tRNA translocation induced by EF-G

To illustrate the applicability of our morphing method on a large system, we generated an atomistic trajectory of tRNA translocation in the 70S bacterial ribosome, catalyzed by elongation factor G (EF-G). This system comprises more than 150 000 non-hydrogen atoms. Despite its large size, the conformational rearrangements of the ribosome can be modeled accurately with surprisingly few flexible residues. In prior work we showed that flexibilizing small documented (7,52) hinge regions at the neck, base of the beak, and spur was sufficient to recapitulate an alternate conformation of 16S, within 0.8 Å RMSD, when driven by flexible fitting forces. In a random thermal exploration, this 16S model repeatedly recapitulated three alternate conformations, within 2 Å RMSD. tRNAs flexibilized much as in the current work were fitted to density maps corresponding to three extreme conformations, and recapitulated all three conformations, with Cross Correlation Coefficient of 0.9 (21). This success is due to the considerable extent to which rearrangements can be described as domain motions.

**Figure 4.**
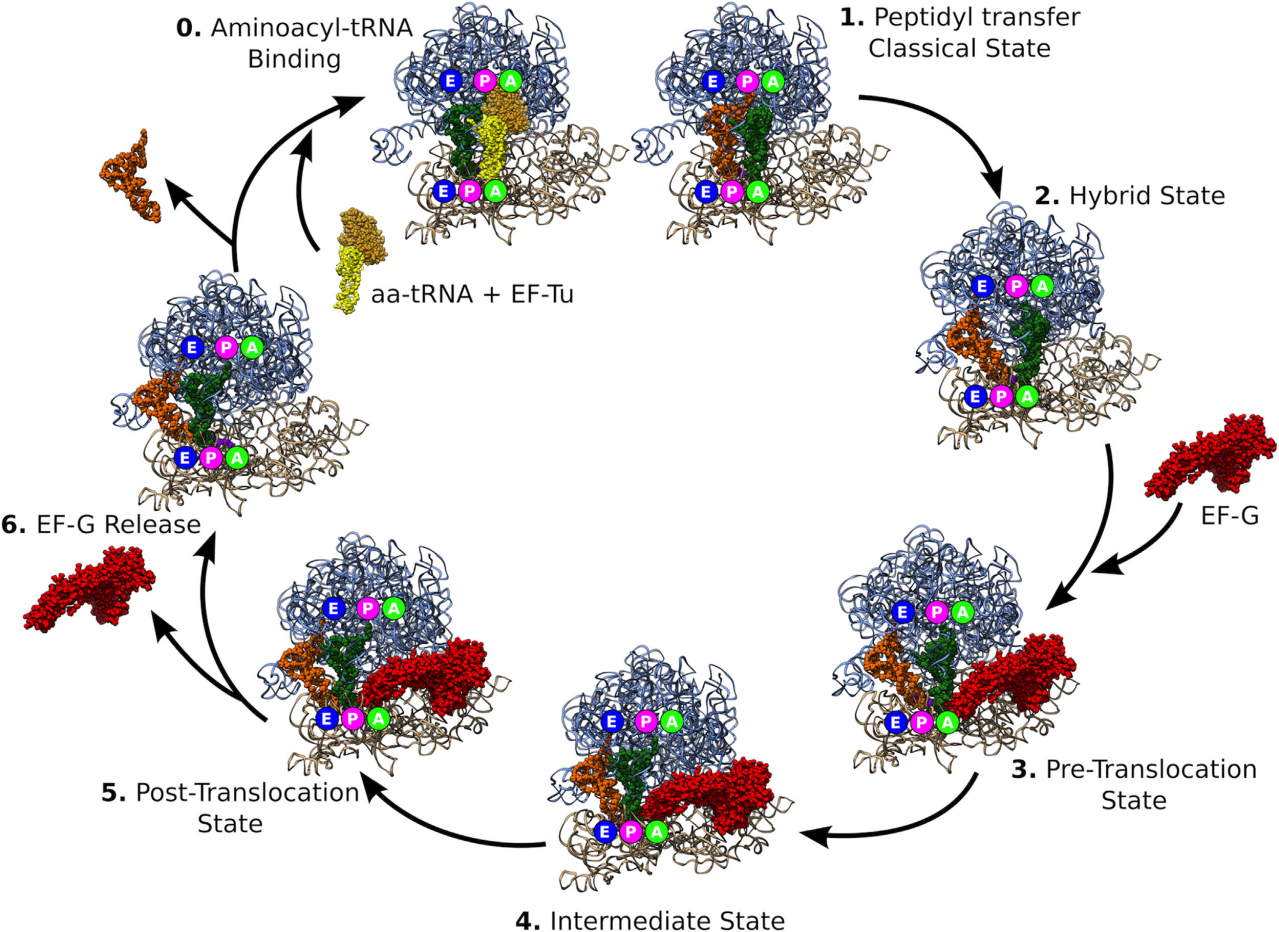
tRNA translocation cycle in the 70S bacterial ribosome. tRNA in green is moving from A site to P site. tRNA in orange is moving from P site to E site and then ejected after step 6. EF-G, in red, binds the ribosome between steps 2 and 3 and is released after step 5. After translocation, a tRNA, in yellow, is brought into the A site and a new cycle begins.

During protein synthesis, tRNAs translocate from A (aminoacyl) to P (peptidyl) to E (exit) site (Figure 4), traversing >100 Å within the ribosome (53). Translocation occurs following peptide bond formation between the peptidyl moiety of peptidyl-tRNA in the P site and aminoacyl moiety of aminoacyl-tRNA in the A site. Translocation proceeds in two global steps. The first step is the displacement of the acceptor ends of the tRNAs in A and P sites to the P and E sites of the large 50S subunit, while the anticodon stem loops (ASL) remain in the A and P sites on the small 30S subunit, resulting in the A/P and P/E hybrid states. Formation of the tRNA hybrid states is coupled with intersubunit rotation, during which the small subunit rotates clockwise relative to the large subunit by up to 12° (54–57). In the second step, which occurs during the reverse, counterclockwise, rotation of the small subunit, EF-G triggers a concerted movement of the mRNA and tRNAs from the A/P and P/E states into the P and E sites, respectively, thus completing translocation of tRNAs into the classical P and E sites on both subunits (58).

Recent structural studies yielded crystallographic and cryo-EM structures of several intermediates of EF-G-induced translocation. These intermediates include globally different conformations of the ribosome, in which the small subunit rotates relative to the large subunit (59). Intrasubunit conformational changes are also involved in translocation, including the movement of the head of the small subunit (60,61) and the L1 stalk of the large subunit (62,63). The transition between these globally very distinct intermediates, including recently reported structures, (64) has not previously been visualized (65). We have simulated the detailed transition by morphing between published structures representing successive translocation states. In these structures, the small subunit, tRNAs and EF-G have been captured in distinct conformations.

**Figure 5.**
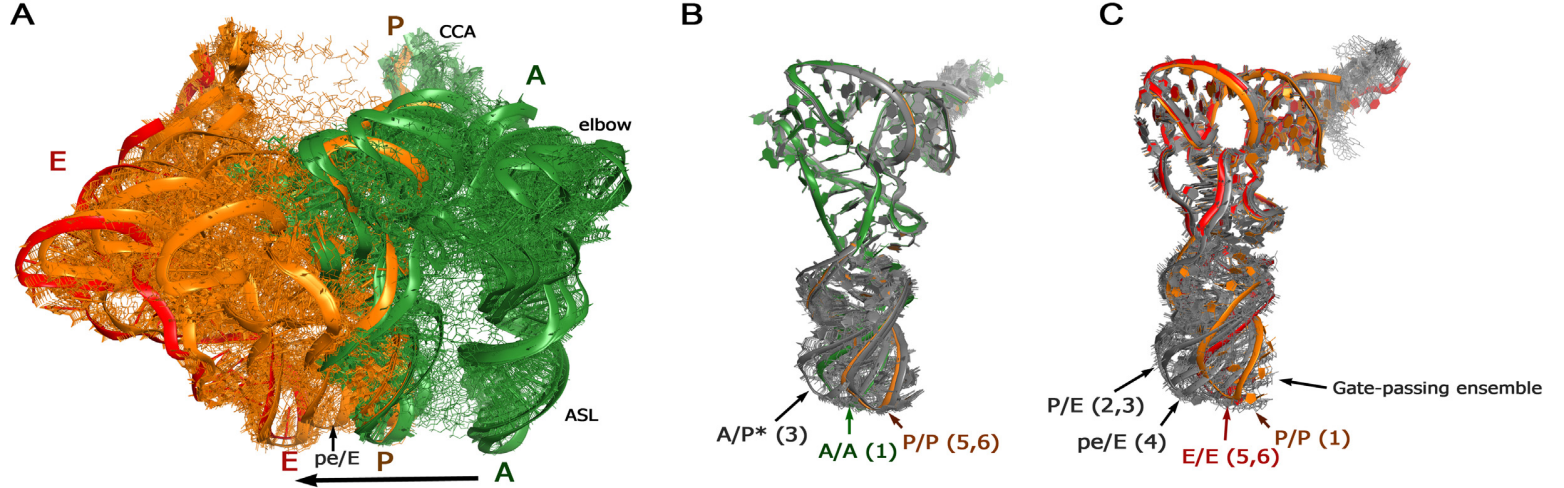
Dynamics of tRNA in the course of translocation between the A (green), P (orange) and E (red) sites. (**A**) Experimental structures are shown with thick ribbon, morphed intermediates are shown with thin lines. The arrow shows the direction of tRNA translocation. (**B**) Superposition of tRNAs demonstrates large intramolecular motions of the anticodon stem loop for the tRNA translocating from the A to P sites. Superposition was performed in Pymol; the anticodon loop residues (nt 30–40) were exluded from superposition. (**C**) Superposition of tRNAs (excluding the anticodon loop, residues 30–40) demonstrates large intramolecular motions of the anticodon stem loop for the tRNA translocating from the P to E sites. The anticodon stem loop (ASL), elbow and 3′-terminal nucleotides CCA are labeled.

**Figure 6.**
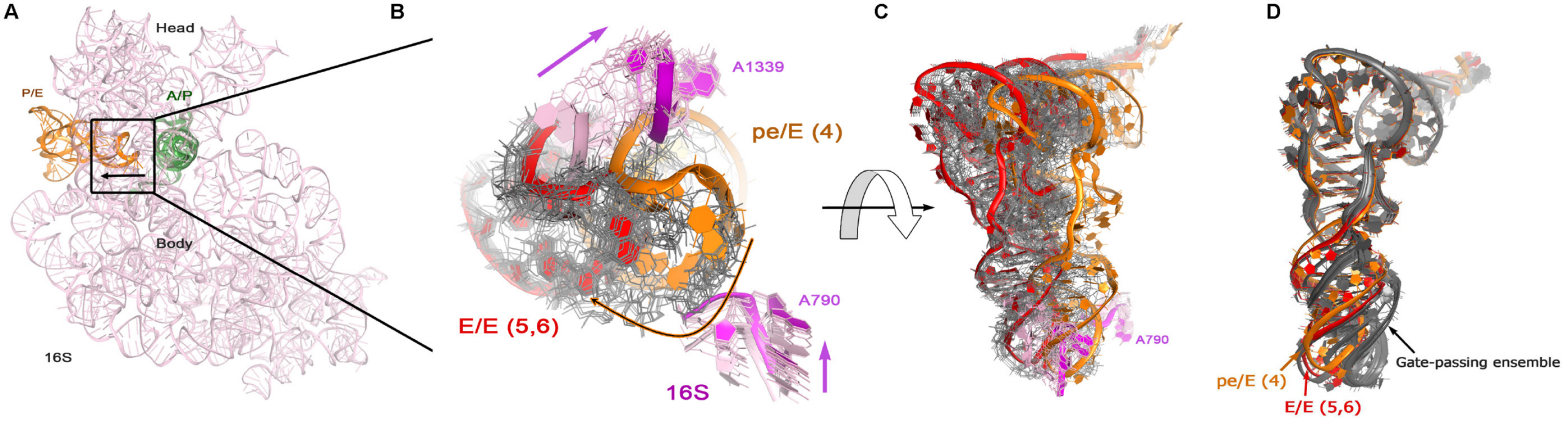
The dynamics of tRNA passing through the gate between the P and E sites, formed by the 16S ribosomal RNA loops of the head and body of the small subunit (residues 1338–1342 and 789–791, respectively). (**A**) The solvent-side view of 16S ribosomal RNA, with pre-translocation tRNAs on the opposite side. The arrow shows the direction of translocation. (**B**) Close-up view of the anticodon-stem loop (ASL) of the tRNA passing the gate, between the pE/E (orange, structure 4) and E/E states (red, structures 5,6). The pink arrows show the direction of the movement of 16S rRNA gate-forming loops coupled with tRNA translocation, the trajectory of which is shown with the orange arrow. Starting and ending states are experimental structures and are shown as thick ribbon, the morphed intermediates are shown with thin lines. (**C**) The view of full tRNA transiting from the P to E site. The colors are as in panel B. Panels A–C were obtained from the morph between 70S structures excluding ribosomal proteins. (**D**) The view of full tRNA passing through the gate as the tRNA transitions between the pE/E (orange) and E/E (red) states, morphed using 70S structures including ribosomal RNA and proteins. Intermediate structures are shown in gray.

The morphing has been calculated between the following experimentally determined structures (Supplementary Table S1). (i) Classical-state non-rotated ribosome representing a state immediately following peptide bond formation, with peptidyl-tRNA in the A site and deacyl tRNA in the P site (66). (ii) Spontaneously formed rotated ribosome, with peptidyl- and deacyl-tRNAs in the hybrid A/P and P/E states, respectively (59). (iii) Pre-translocation EF-G-bound ribosome, with peptidyl- and deacyl-tRNAs in the hybrid A/P* and P/E states, respectively (59). The A/P* state differs from the A/P state in that the elbow of the A/P* tRNA is positioned closer to the P site. Thus, the A/P* tRNA is closer to the fully translocated P/P (or P) state than the A/P tRNA. (iv) Intermediate pre-translocation EF-G-bound ribosome, with tRNAs located between the hybrid and classical states (67). Here, a distinct intermediate state of tRNA was observed, in which the acceptor arm of the tRNA is in the 50S E site, while the anticodon stem loop is between the P and E sites on the 30S subunit (Figure 5A). As discussed below, the ASL is kept from moving into the E site by a ‘gate’ formed by 16S ribosomal RNA (Figure 6). In this experimentally observed structure, the translocating tRNA is closest to the E site, thus the intermediate is named the pe/E tRNA (Figures 5 and 6). (v) Post-translocation non-rotated EF-G-bound ribosome, with fully translocated peptidyl- and deacyl-tRNAs in the classical P and E sites, respectively (68). (vi) Post-translocation non-rotated ribosome in the absence of EF-G, with translocated peptidyl- and deacyl-tRNAs in the classical P and E sites, respectively (66). We used the 3.3 Å crystal structure from *T. Thermophilus* (66) (PDB IDs: 2WDG and 2WDI) as a base structure. Each subsequent morph used the resulting structure of the previous morph as starting point.

As the large-scale conformational changes are driven by ribosomal RNA, in a first morph we have excluded all ribosomal proteins other than EF-G. As described in a previous paper (36) and Supplementary Table S2, we allowed flexibility at the base of the neck and the beak of 16S ribosomal RNA (rRNA), the base of L1 stalk and the A-site finger (H38) on 23S rRNA, and at the base of the anticodon stem-loops of the tRNAs. Collision-detecting spheres (17) around these zones prevent clashes and preliminary runs were used to add additional spheres where the acceptor ends get close to 23S rRNA during their displacement (35). The initial model of mRNA came from structure 1. We made mRNA entirely flexible and imposed Watson-Crick interactions between codon bases and their respective anticodon bases on the tRNAs. The initial EF-G structure came from the structure of pre-translocation state bound with A/P* and P/E tRNAs (59). EF-G was made flexible between domains II and III and between domains III and IV to allow previously mentioned structural changes to pass from the pre- to post-translocation conformations (59). We placed EF-G at distance from the ribosome after steps 2 and 5 to mimic its binding, during transition from step 2 to step 3, and release, during transition from steps 5 to 6.

For each morph, the final structure was first rigidly aligned to the initial structure based on the 16S RNA, using Chimera's MatchMaker tool. Then each chain of the initial structure was morphed to the corresponding one on the final structure using MMB's *gappedThreading* command, which automatically applies springs connecting corresponding atoms based on a gapped alignment (47). Only the mRNA was morphed using the *threading* command (which uses a manual alignment) to ensure correct translocation of the codon bases. We stopped the simulations when the energy difference between two consecutive frames was below 50 kJ/mol during five frames. Visual inspection validated the resulting structures.

Each morph included between 144 791 and 155 378 moving atoms and took 20 to 43 min to converge on a laptop. The longer morphs are occurring during translocation completion (from state 4 to step 5), and after translocation (from step 5 to step 6). The 5′ regions of mRNAs of the target structures are generally not modeled in the mRNA exit tunnel beyond the E site codon, whereas our translocated mRNA is ending one codon further in the exit tunnel. Finding an energetically stable conformation for these dangling residues accounts for part of the added cost.

To demonstrate MMB's ability to handle the entire ribosome, we generated new trajectories of the translocation completion with most of the proteins available in the crystal structures of states 4 and 5. Some proteins where not included because of their absence in one structure (L1, absent from PDB structure 3J5N used for state 4), or due to incompletely-resolved structures (l, m, U). In order to keep runtimes short, most proteins were kept rigid and welded to the rRNA of their subunit. Only protein S7, located very close to the E site was made partly flexible in an attempt to accommodate the translocation of the tRNA from the P site to the E site. The threading force constant *F* for the P-site tRNA was increased from 30 to 60 to compensate transient contacts with S7. With *F = 30*, the tRNA became stuck in the gate. This system is composed of 249 313 atoms and morphing converged in about 45 minutes.

Lastly, we wished to demonstrate MMB's ability to recapitulate known intermediates not only for the proteins in the Weiss and Levitt dataset, but also of the ribosome. With that in mind, we morphed from state 1 directly to state 3, without using the state 2 structure. Our trajectory partially recapitulated state 2, despite the fact that the latter lies off the linear interpolation pathway (Figure 8).

### RESULTS AND DISCUSSION

Viewers such as VMD and Chimera have matured to the point that rendering and basic geometric operations can easily be done on a laptop, even for the largest macromolecular complexes. Similarly, tasks such as flexible fitting, (21) homology modeling, (24) prediction of ΔΔG of protein-protein binding, (37) and macromolecular folding, (26) have also become efficient enough to run on a laptop (30). And yet, such operations have remained out of reach of many non-specialists due to the limitations of a command-line interface. In this work we bridged this gap, adding the mentioned and many other modeling capabilities to Chimera. With this interface, users control MMB visually and interactively. Users can input commands by clicking directly on atoms as well as through intuitive forms which present the user with available options. The simulation can be started, paused, and restarted.

MMB-GUI is a versatile tool that can be used to economically morph large macromolecular complexes, which are heterogeneous in sequence and even chain count. The novel morph method accurately recapitulates known intermediates for such structurally distinct proteins as RNase III, ribose-binding protein, myosin, Ca^2+^ ATPase and 5′-Nucleotidase, which form the benchmark set of Weiss & Levitt (45). While our method was globally better than all others benchmarked, (45) for three of the proteins (5′ Nucleotidase, Myosin, and Ribose Binding Protein) we obtained a high improvement score (Figure 3) and low RMSD (Supplementary Figure S3). These are domain hinge bending proteins, with clear domain boundaries and few intradomain rearrangements; (39) in fact RBP appears in the Hinge Atlas Gold benchmark dataset (69). They are thus highly amenable to our multiscale method.

The case of Ca^2+^ ATPase bears discussion. In order to follow a uniform protocol for all five test cases, we used a flexibility scheme that assumed a domain hinge bending motion, with hinges predicted using HingeMaster, (50) and obtained a minimum RMSD vs. final of a rather high 5.13 Å. However inspection of the morph reveals that Ca^2+^ ATPase contains multiple domains with unclear boundaries, intra-domain rearrangements, and changes of secondary structure. In short, it does not meet the definition of domain hinge bending, (39) and for this reason is less amenable to a multiscale treatment. Flexibilizing residues 42–47, 57 -59, 80–84, 112–114, and 122–126 in order to permit more of these rearrangements decreased the the RMSD vs. final to 4.15Å, however RMSD vs. intermediate actually increased to 9.09 Å.

The ability to recover intermediates and economically model large ribonucleoprotein complexes with heterogeneous chain counts, especially in cases that involve domain motions, makes our morphing method well suited for modeling ribosomal rearrangements (21). We created a didactic movie of EF-G-mediated translocation of tRNA during translation, allowing us to visualize structural intermediates between static experimentally determined structures.

The intermediate structures produced by morphing, provide interesting suggestions regarding the dynamics of the tRNAs during translocation. Here, we discuss two observations (1). Superposition of the tRNA intermediates suggests that the A- and P-tRNAs undergo similar motions during translocation to the P and E sites, respectively, despite the facts that the movements of two tRNAs are not synchronized and the tRNAs move in different trajectories (Figure 5). Specifically, as the A-tRNA moves to the neighboring P site its CCA 3′-end and elbow travel up to 10 and 40 Å, respectively, whereas the movement of the P-tRNA into the E site requires that the CCA end and the elbow traverse ∼40 and 60 Å, respectively (Figure 5A). Yet, the domain motions of the anticodon-stem loop (ASL) relative to the rest of the tRNA are similar in that residue 34 at the tip of the anticodon loop sweeps the large conformational distance of ∼14 Å in the course of A->P translocation (Figure 5B) and ∼17 Å during the P->E translocation (Figure 5C). The A->P conformational states are represented by the intermediates between the two extreme A-tRNA conformations observed experimentally in the EF-G bound state, namely the A/P*pre-translocation (59) and the P/P post-translocation states (70). This phenomenon easy to visualize in the morph, but can also be observed by toggling (i.e. leaving out the grey structures in Figure 5B and C). By contrast, the morphing of the P->E translocating tRNA creates a previously unobserved ensemble of intermediates, which sample one of the ‘extreme’ states of the ASL motion relative to the rest of tRNA (Figure 5C). This sampling occurs in the course of movement through a narrow channel (gate) between the body and head of the small ribosomal subunit, between residues 790 and 1339, respectively (Figure 6). In the non-rotated or partially rotated ribosomes, these residues were proposed to block the movement of the ASL from the P to E site due to insufficient space (∼13 Å wide) for the ASL transit (71). A recent structure of the ribosome in the intermediate state of translocation (structure 4) revealed that the channel widens to more than 20 Å due to swiveling of the head of the small subunit, (67) in principle allowing for the ASL transit. This structural information is essential to our analysis; the translocation completion morph did not converge if structure 4 was left out. The ASL of the translocating pE/E tRNA, however, is still kept from entering the gate and maintains the contacts with the 30S P site. Thus, it remains unclear whether the experimentally observed opening of the gate is sufficient for tRNA passage to the E site or additional changes in the tRNA and/or 30S subunit are necessary. Our morphing suggests that the movement of the tRNA through the gate is coupled with ‘untwisting’ of the ASL relative to the D stem (Figure 6B and C). In this conformation, the ASL is rotated by nearly 30^o^ relative to that in the pE/E state (Figure 5C), emphasizing that substantial conformational rearrangements in the tRNA are required to pass the channel if no further head swivel occurs.

**Figure 7.**
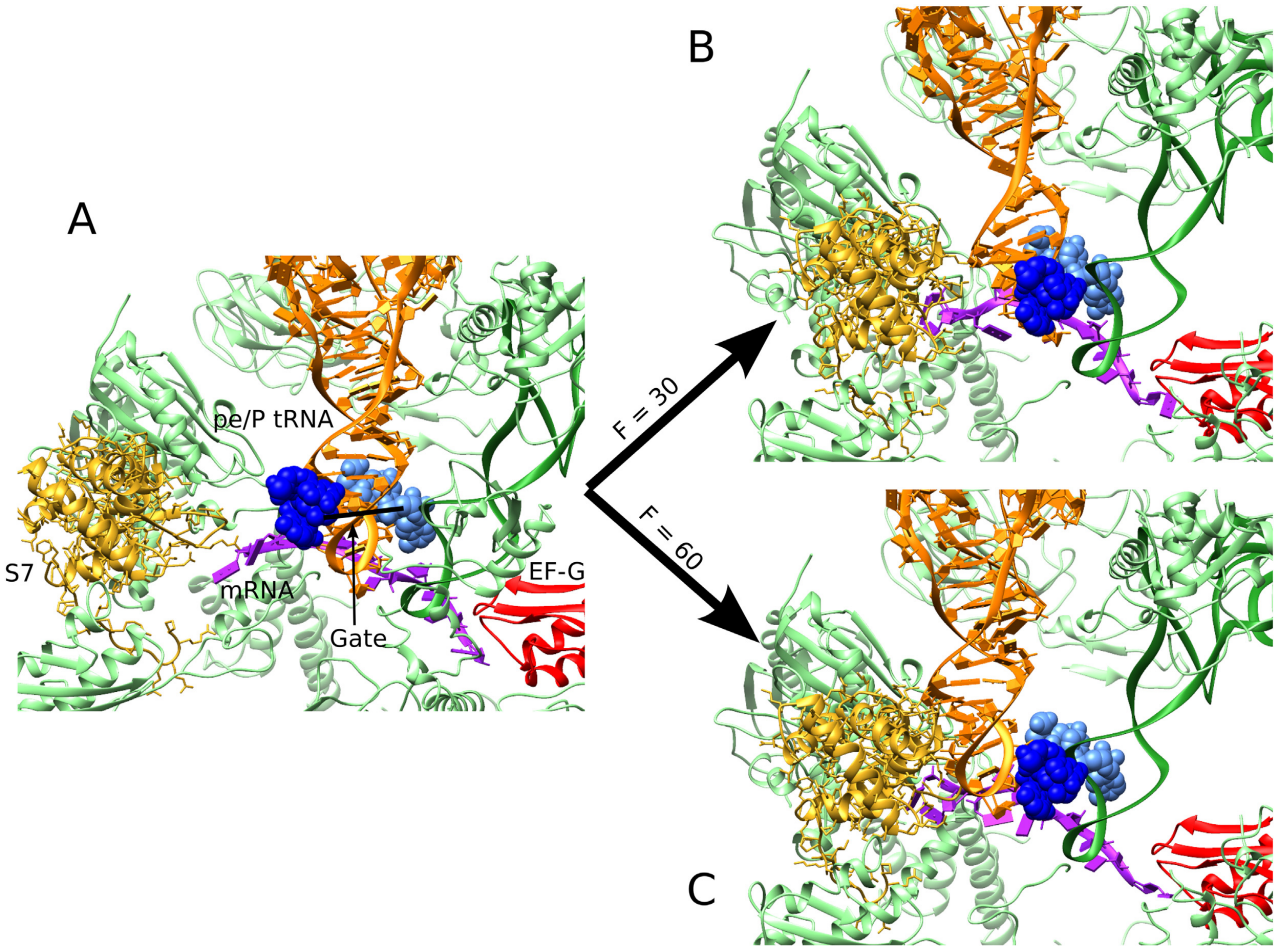
Two different outcomes when recapitulating the ribosomal translocation by morphing with ribosomal proteins. (**A**) Original conformation in the intermediate state (step 4). For clarity, protein S13 and rRNAs were hidden to the exception of the gate residues of 16S (789–791 and 1338–1342). (**B**) Resulting conformation when applying a threading force Constant (*F*) of 30 to the tRNA in the pe/E conformation. While the gate is shifted due to 16S head rotation, the tRNA stays in a pe/E state. (**C**) Resulting conformation when applying a threading force constant (*F*) of 60 to the tRNA in the pe/E conformation. In this case, the tRNA is able to pass the gate and adopt the E conformation.

As mentioned, the above was done with no ribosomal proteins except EF-G. To test the role of ribosomal proteins in translocation, we then generated a morph which included all proteins. In a first attempt, we couldn't observe a complete translocation of the tRNA from the P site to the E site. While the acceptor arm and the elbow show a proper transition, the ASL seems unable to pass through the gate and is stuck in the middle (Figure 7B). Nonetheless, the target structure (PDB file 2WRI) shows clearly that protein S7 can accommodate the presence of a tRNA in the E site. Since S7 is located very close to the E site, it appears to transiently repel the ASL in the morph. Moderately increasing the threading force for the tRNA resulted in translocation (Figure 7C). Here, a β-hairpin of S7 (aa 77–84) engages with the translocating tRNA immediately following the passage of the gate, in keeping with the role of this S7 region in maintaining the open reading frame (72). The tRNA rearrangements with proteins (Figure 6D) resemble those in the morph performed in the absence of ribosomal proteins (Figure 5B and C), as they include the widening in the anticodon stem loop. Thus, the morphing with ribosomal proteins confirms the ‘untwisting’ of the tRNA during the gate passage.

**Figure 8.**
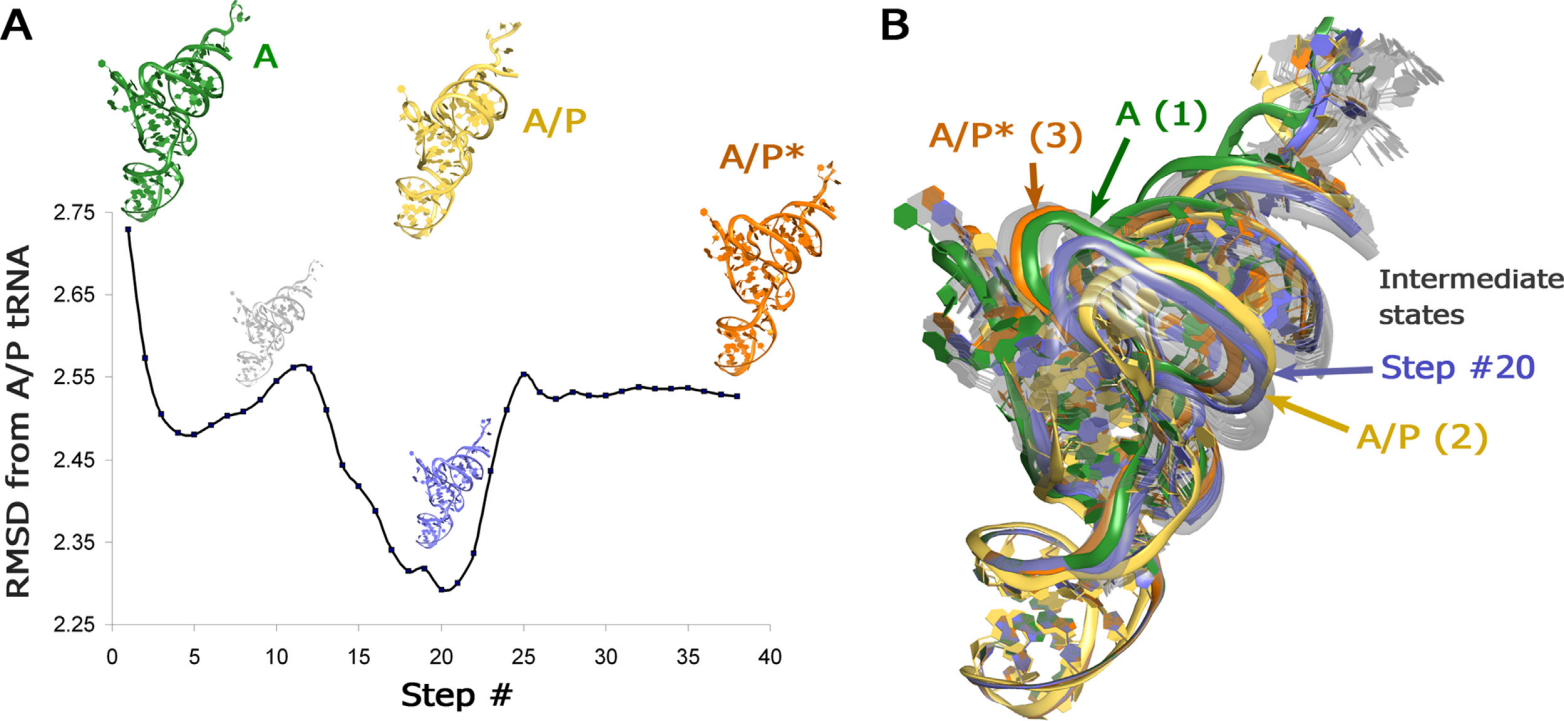
Morphing of tRNA translocation from state 1 (A-tRNA, PDB 2WDG, 2WDI) to state 3 (A/P* tRNA, PDB 3J5W, 3J5X) recovers intermediate structures that resemble the omitted state 2 (A/P tRNA, PDB 3J5T, 3J5U). (**A**) All-atom root-mean-square difference of morphed A-site tRNA structures from the intermediate A/P tRNA, which was not used in the morph. (**B**) Structures of the starting A-tRNA (green), final A/P* tRNA (orange) and intermediate morph structures (gray), including intermediate structure #20 (blue), which most closely resembles the omitted state-2 A/P tRNA (yellow). The tRNA structures were aligned by superposition of the anticodon stem loop regions (residues 26–44).

To explore the predictive potential of morphing, we have attempted to recapitulate an experimentally determined intermediate structure, by omitting it from a simulation. To this end, we have performed morphing between ribosome structures 1 and 3, leaving out structure 2. Structure 2 represents an intermediate state of the A-site tRNA translocation (A/P), in which the acceptor arm of the tRNA is shifted toward the P site of the 50S subunit, resulting in a conformation that is different from both the classical A-site tRNA (1) and the EF-G-bound A/P* tRNA (3) structures. In our simulation, the intermediate states in the middle of morphing closely resembled the omitted A/P structure of tRNA (Figure 8). This is remarkable because the intermediate A/P structure substantially deviates from a linear trajectory, in that the elbow shifts in the direction opposite to its final destination (Figure 8B). Thus, the positions of the elbow and the acceptor arm of the A/P tRNA relative to the anticodon stem loop are better represented by the morphing intermediates than by the starting or ending structures. This unbiased test demonstrates that despite being a highly restrained simulation, with only two rigid groups defining the conformational freedom of tRNA, the morph may reveal intermediate conformations that globally resemble existing structures.

Morphing is unlikely to have the predictive power of MD, as we have limited the flexibility and physics of the system. Despite these limitations, our observations suggest that the computationally economical GUI-controlled morphing may yield new functionally relevant information that merits validation and detailed examination with further computational (e.g. MD) and experimental studies.

## AVAILABILITY

Input files for each morph, the final trajectory and video files are freely available online (https://simtk.org/home/efgtranslocat). MMB 2.15 documentation, source code, and binary packages for OSX and Linux (with a Windows release planned) are freely available online (https://simtk.org/home/rnatoolbox).

## Supplementary Material

SUPPLEMENTARY DATA
