# Supplementary material for: MMB-GUI: a fast morphing method demonstrates a possible ribosomal tRNA translocation trajectory
Source: Nucleic Acids Res. 2015 Dec 15;44(1):95–105. doi: 10.1093/nar/gkv1457 (PMC4705676; doi:10.1093/nar/gkv1457)
Supplement: SUPPLEMENTARY DATA [file supp_44_1_95__index.html]

MMB-GUI: a fast morphing method demonstrates a possible ribosomal tRNA translocation trajectory — MMB-GUI: a fast morphing method demonstrates a possible ribosomal tRNA translocation trajectory — SUPPLEMENTARY DATA 

# MMB-GUI: a fast morphing method demonstrates a possible ribosomal tRNA translocation trajectory

## SUPPLEMENTARY DATA

- SUPPLEMENTARY DATA
- SUPPLEMENTARY DATA
- SUPPLEMENTARY DATA
