# Supplementary material for: MMB-GUI: a fast morphing method demonstrates a possible ribosomal tRNA translocation trajectory
Source: Nucleic Acids Res. 2015 Dec 15;44(1):95–105. doi: 10.1093/nar/gkv1457 (PMC4705676; doi:10.1093/nar/gkv1457)
Supplement: SUPPLEMENTARY DATA [file supp_gkv1457_nar-00391-n-2015-File010.docx]

# Supplementary information

## Implementation

### Fast communication between MMB and Chimera

Since version 2.15, MMB includes a Graphical User Interface, or GUI, implemented as a plugin for Chimera. MMB is written in C++, while Chimera uses Python to implement its high-level features, user interface and plugins. Using the ctypes library, we created a python interface for MMB, which accesses the relevant methods and data structures. The resulting Python module, pyMMB, can be used in any Python script and thus in a Chimera plugin. Simulations are launched in their own thread, which allows the use of Chimera’s UI concurrently, maintaining the performance of the MMB C++ library. An asynchronous callback allows fetching data and structures while the simulation is running.

### A GUI built on the Chimera platform allows users to intuitively control all MMB features

Chimera uses Tkinter as its GUI system. Our plugin is built on a Model-View-Adapter model. pyMMB is the model, Chimera is the view and a intermediate class is used to adapt pyMMB data structures into objects easily used by Chimera and Tkinter (Figure S1).

The main objective of the GUI is to offer an interactive and user-friendly way to input MMB commands and check the output of the simulation. Tabs are used to group these commands by features (Figure S2). Three steps must be followed to run a simulation.

The first step is to initialize MMB with biopolymers, represented by a type, chain ID, sequence and structure. Users have several options to do so, as follows. 1. Use a form to generate new polymers. Users specify the type, chain ID, first residue number and the sequence; a structure will be generated with default coordinates. 2. Open one or more PDB files from which the chains, sequences and structures will be extracted. 3. Import chains and structures from models already opened in Chimera. 4. Use the command line to type an MMB biopolymer command. 5. Read a commands file containing such commands. Users can then review and edit polymers in the Input tab. When ready, the user clicks on the *Load* button. The program instantiates all the chains specified, determines their initial conformation, and sets atom names consistent with the PARM99 potential.(Cornell  Cieplak, P., Bayley, C. I., Gould, I. R., Merz, K. M., Ferguson, D. M., Spellmeyer, D. C., Fox, T., Caldwell, J. W. & Kollman, P. A., 1995) This model is then loaded in Chimera for visualization and manipulation.

The second step is to set the flexibility, constraints, forces, and physics zones (Dourado & Flores, 2013). Each MMB command is represented by a line of drop-down lists and other widgets allowing the user to easily choose chain IDs, residue IDs etc. Commands are validated when added or updated and can be rejected, yielding informative error messages. Chimera then displays an intuitive visual representation of the commands; likewise command parameters (e.g. atoms involved in an interaction, residues of altered flexibility, etc.) can be specified using Chimera selection features (Figure 1 in main text). At all these stages, users can save and load MMB commands from a file or enter individual commands in the provided command line.

The third step is to run a simulation. The simulation tab presents some commonly used parameters, like reporting intervals, temperature *etc*. All other parameters are concurrently accessible from a separate window. Initialization of the simulation takes into account every entered command and sets up the internal coordinate topology. As the calculation of molecular trajectories proceeds, every computed frame is being loaded into a Trajectory panel, allowing an easy review of the simulation. The *Run* button launches the computation for the number of intervals entered by the user. After an interval is computed, a text display is updated with the current energy values and other information, and the last frame is added to the trajectory in Chimera. Once a run is over, users can change the number of intervals and launch a new run with a click, starting from the last frame, or another as chosen in the Trajectory panel. Once the user is satisfied with the simulation, it is possible to save the intermediate structural models using Chimera’s standard interface.


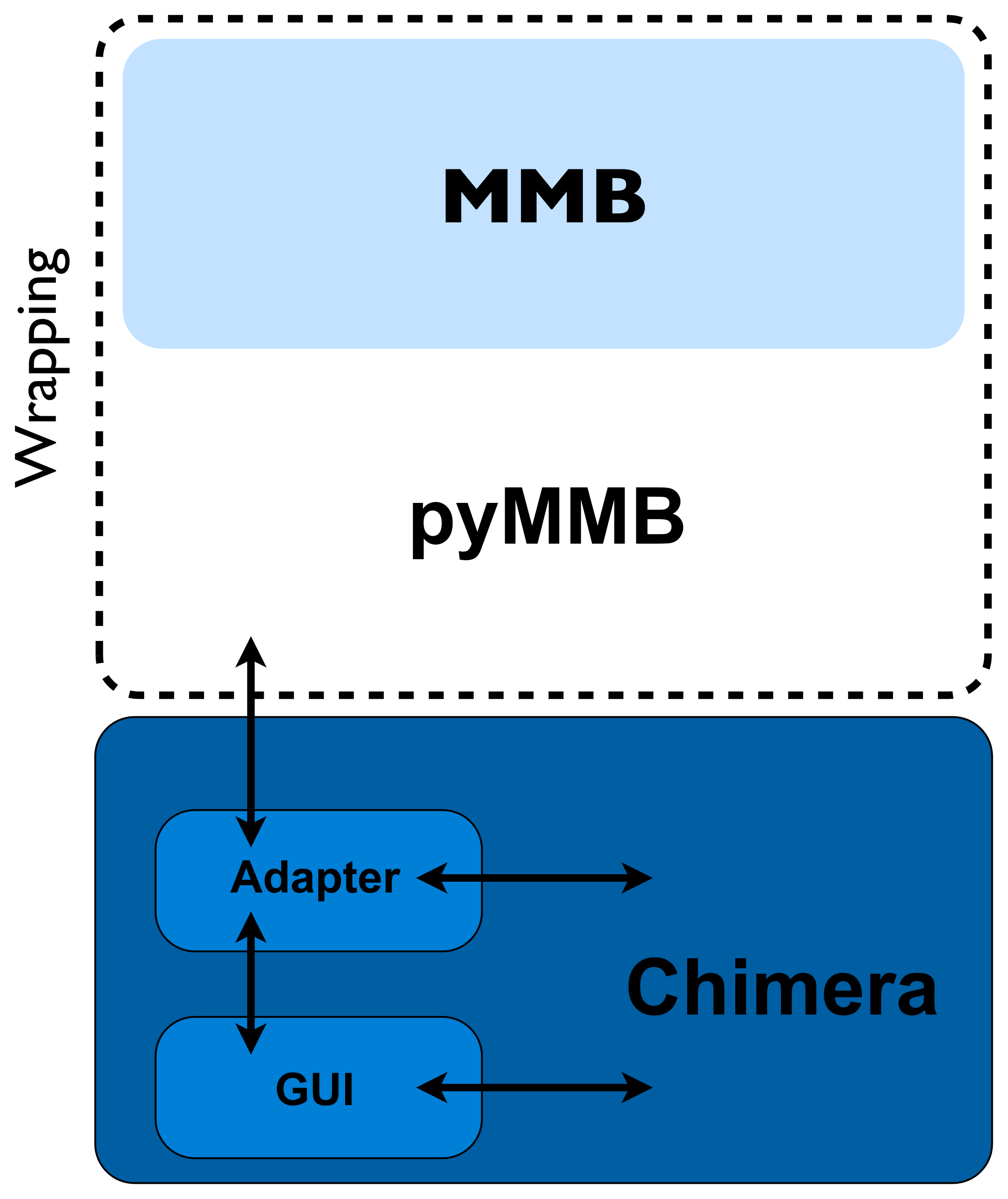


Figure S1. Interaction between MMB and the GUI plugin for Chimera.


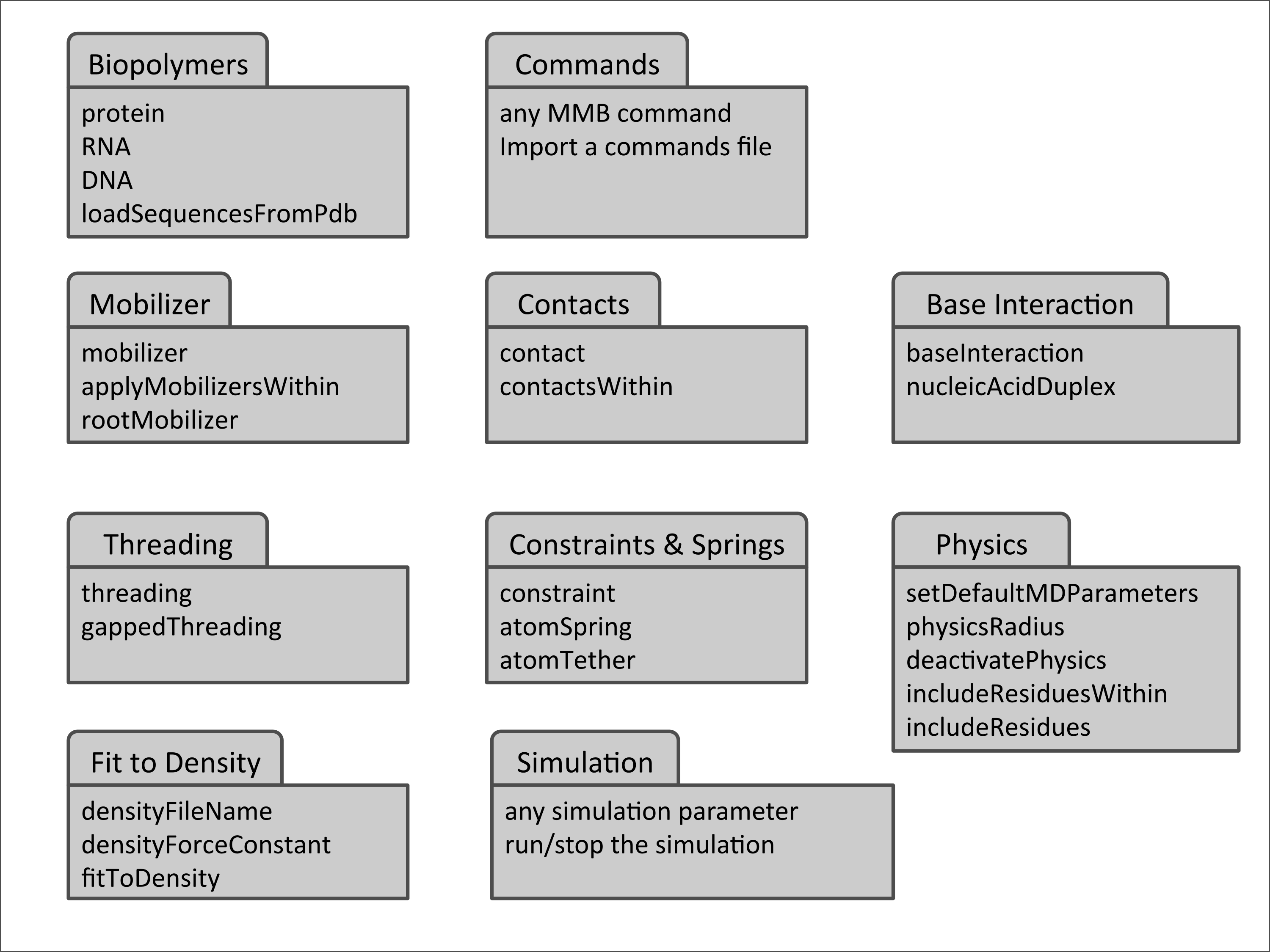


Figure S2. Summary of MMB’s commands available in the GUI. Different kinds of features are organized with tabs. A command line in the *Commands* tab allows loading any command.


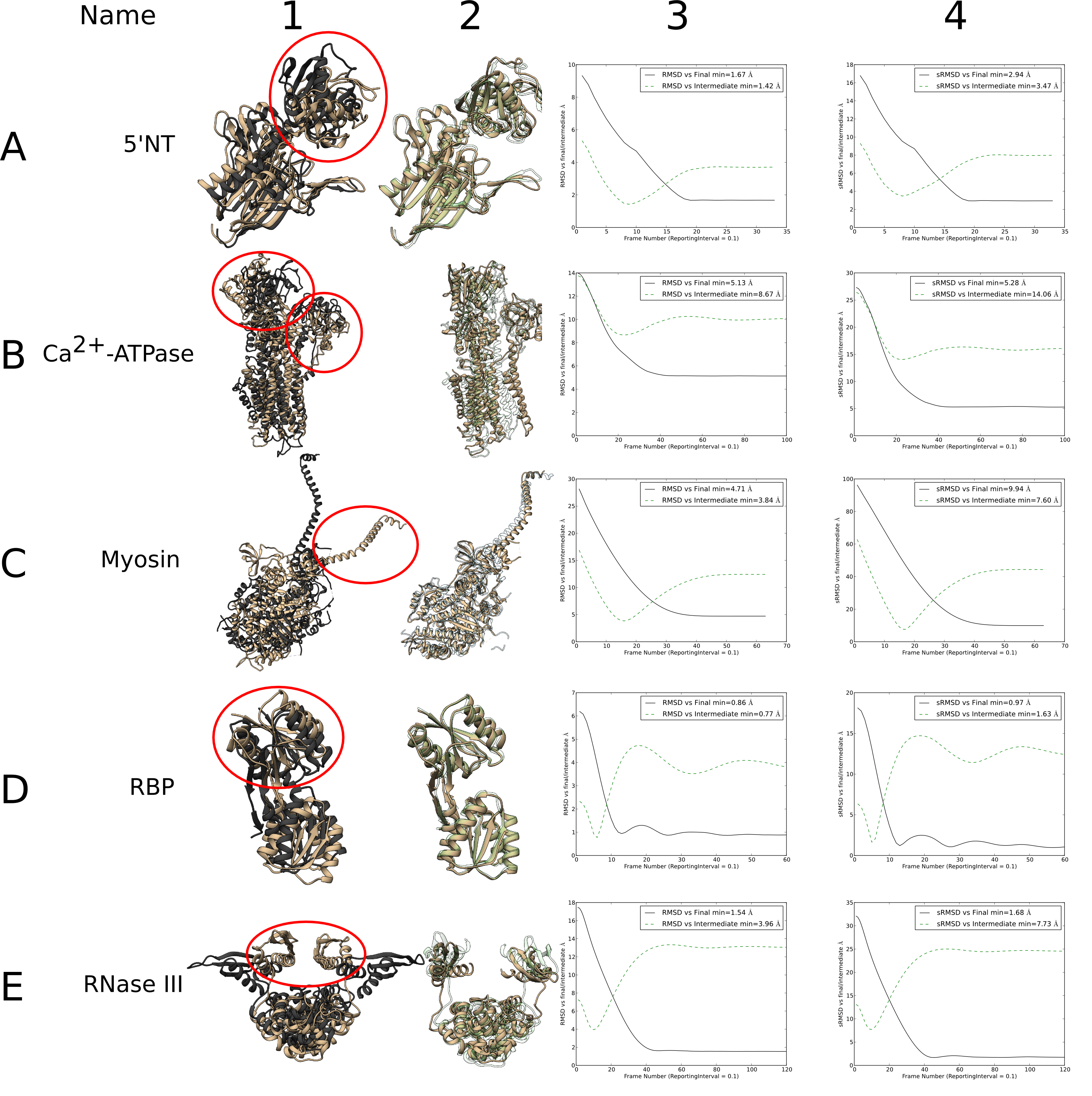


Figure S3. Morphs of various proteins from an open to a closed conformation with recapitulation of an intermediate structure. In each case, the intermediate structure has been elucidated experimentally (Weiss & Levitt 2009). Column 1: initial structure (tan) superposed on final structure (black). Mobile domains for sRMSD calculations are indicated with red ovals. Column 2: Superposition of the closest interpolated structure (tan) to the known intermediate (green). Column 3: Cα RMSD of the morphed vs. final structure and intermediate structures. Column 4: Cα sRMSD of the morphed vs. final structure and intermediate structures.  sRMSD is determined by first aligning the two structures based on an arbitrarily chosen fixed domain (here, the larger of the two), then computing RMSD of the mobile domains. (Maiorov & Abagyan, 1997)

Table S1 Key frames corresponding to each state and experimental structures used to generate the trajectory

| Step | Frame | Ribosome State | tRNA sites | Structures | Organism |
| --- | --- | --- | --- | --- | --- |
| 1 | 1 | Classical state  EF-G free  Pre-translocation | A  P | 2WDG (Voorhees, Weixlbaumer, Loakes, Kelley, & Ramakrishnan, 2009)  2WDI | *T. Thermophilus* |
| 2 | 35 | Hybrid state  EF-G free  Pre-translocation | A/P  P/E | 3J5T (Brilot, Korostelev, Ermolenko, & Grigorieff, 2013)  3J5U | *E. Coli* |
| 3 | 58 | Hybrid state  EF-G bound  Pre-translocation | A/P*  P/E | 3J5W (Brilot et al., 2013) 3J5X | *E. Coli* |
| 4 | 77 | Intermediate state  EF-G bound | Inter. P  Inter. E | 3J5N (Ramrath et al., 2013) 3J5O | *E. Coli* |
| 5 | 92 | Classical state  EF-G bound  Post-translocation | P  E | 2WRI (Gao et al., 2009) 2WRJ | *T. Thermophilus* |
| 6 | 107 | Classical state  EF-G free  Post-translocation | P  E | 2WDG (Voorhees et al., 2009)  2WDI | *T. Thermophilus* |

Table S2 – Flexible residues in the ribosome, tRNAs and EF-G during morphing. Residue numbers follow the numbering of PDB structures 2WDK and 2WDL (Voorhees et al., 2009).

| Subunit | Residues | Description |
| --- | --- | --- |
| 16S | 926, 1391 | Neck |
| 16S | 995, 1043, 1044 | Base of beak |
| 23S | 2092, 2093, 2197 | Base of L1 stalk |
| 23S | 848, 928 | Base of A-finger (H38) |
| tRNAs | 72-76 | Acceptor terminus |
| tRNAs | 26, 44 | Anticodon stem (during hybridization) |
| mRNA | All |  |
| EF-G | 406 to 416 | Linker between domains II and III |
| EF-G | 485 to 490 | Linker between domains III and IV |

## References

Brilot, A. F., Korostelev, A. A., Ermolenko, D. N., & Grigorieff, N. (2013). Structure of the ribosome with elongation factor G trapped in the pretranslocation state. *Proceedings of the National Academy of Sciences of the United States of America*, *110*(52), 20994–9. doi:10.1073/pnas.1311423110

Cornell  Cieplak, P., Bayley, C. I., Gould, I. R., Merz, K. M., Ferguson, D. M., Spellmeyer, D. C., Fox, T., Caldwell, J. W. & Kollman, P. A., W. D. (1995). A second generation force field for the simulation of proteins, nucleic acids and organic molecules. *Journal of the American Chemical Society*, *117*, 5179–5197.

Dourado, D., & Flores, S. C. (2013). Local physics-based equilibration is key to improved prediction of protein-protein interactions. *Submitted*.

Gao, Y.-G., Selmer, M., Dunham, C. M., Weixlbaumer, A., Kelley, A. C., & Ramakrishnan, V. (2009). The structure of the ribosome with elongation factor G trapped in the posttranslocational state. *Science (New York, N.Y.)*, *326*(5953), 694–9. doi:10.1126/science.1179709

Maiorov, V., & Abagyan, R. (1997). A new method for modeling large-scale rearrangements of protein domains. *Proteins*, *27*(3), 410–424. doi:10.1002/(SICI)1097-0134(199703)27:3<410::AID-PROT9>3.0.CO;2-G [pii]

Ramrath, D. J. F., Lancaster, L., Sprink, T., Mielke, T., Loerke, J., Noller, H. F., & Spahn, C. M. T. (2013). Visualization of two transfer RNAs trapped in transit during elongation factor G-mediated translocation. *Proceedings of the National Academy of Sciences of the United States of America*, *110*(52), 20964–9. Retrieved from http://www.pnas.org/content/110/52/20964

Voorhees, R. M., Weixlbaumer, A., Loakes, D., Kelley, A. C., & Ramakrishnan, V. (2009). Insights into substrate stabilization from snapshots of the peptidyl transferase center of the intact 70S ribosome. *Nature Structural & Molecular Biology*, *16*(5), 528–33. Retrieved from http://dx.doi.org/10.1038/nsmb.1577
